# Meeting of the Fulton County Medical Society

**Published:** 1914-10

**Authors:** R. R. Daly


### FULTON COUNTY MEDICAL SOCIETY.

Regular Meeting October 1, 1914.

Dr. Michael Hoke exhibited slides of "Some Cases Illustrating Methods of Correction of Deformities of the Feet."

Dr. H. I. Battey in discussing the subject said that while he agreed with the greater part of Dr. Hoke's methods, he was convinced that more could be done with manipulation without plastic operation than Hoke seemed to think. The method of Lorenz was still in successful use and when the cases were taken early the ankles and feet could be reshaped many times without the sections.

Dr. J. S. Derr read a paper on the meeting of the American Roentgen Ray Society.

Dr. S. P. Harris said that this specialty is daily increasing in importance and value and that new fields are being opened for the study of the internist as well as the surgeon. In the diagnosis of tuberculosis, the x-Ray has not superseded the other methods of physical diagnosis, but it does delineate effusions more clearly. He also spoke of the "x-Ray Atmosphere," which seems to have a fatiguing effect upon operators long exposed to its influence. This does not refer to burns or other direct exposures.

Dr. G. K. Varden reported a case of a five weeks old child whose nose was stopped with what proved to be a misplaced bicuspid tooth.

REGULAR MEETING OCTOBER 15, 1914.

Dr. George C. Trimble read a paper "Report of Treatment of Pellagra at the Georgia Baptist Orphans' Home." It appears elsewhere in the Journal.

Dr. G. M. Niles said that he had seen over 700 cases of Pellagra and he is ready to agree that the disease comes chiefly among the poorly nourished. There are exceptions appearing among fine specimens of development. It is difficult to prove that Pellagra is infectious, but he would hesitate to live in the house with a pellagrin except under special hygienic precautions. Sambon does not now cling to the fly theory. The stable fly qualifies as a possible carrier of the disease instead of the buffalo fly as at first outlined, but neither has been shown to be the carrier beyond a doubt. As for himself, he admits that he is no longer convinced of the value of the maize theory. The question is sub judice. Allen, of Charlotte, has found a great deal of hookworm with Pellagra.

As to treatment. The disease is of central origin and needs a liberal diet of proteids. The diarrhoea does not contra indicate full feeding of proper foods. Calcium sulfid has not given him good results. Iodide of potassium, Fowler's solution in ascending and reascending doses are of undoubted value. Enough iron can be given in a meat diet. Tanningen will control the diarrhoea. Cacodylate of Soda and Atoxyl are both likely to give eye troubles if continued.

Dr. Roy Blosser said that the prevalence of the disease in Trimble's cases between the ages of 6 and 12 corresponds with the experience among similar children at Jackson where orphans have been investigated.

Dr. Arch Smith said that a diarrhoea with putrid stools should not have proteid diet. Acetone urine occurs and shows the cause of the nervous irritability. It should be removed by carbo hydrate diet and enemata of bicarbonate of soda. The medical treatment by Cacodylate of Soda gr. 3 with Cyanide of Mercury gr. 1-10 injected intravenously twice a week had been of value and not attended with ocular symptoms.

Dr. Hansell Crenshaw asked if mental symptoms had been noted among children. Among adults the mental symptoms do not take any particular form and the cases are classified on other grounds. The fact that they have Pellagra is added to the diagnosis. He agrees that the dermal symptoms are not reflex, but of central origin.

Dr. Trimble closed by saying that there were two or three children of low mentality in the group but they were so anyway. It is important to say that there was no mortality in all his cases there.

Dr. G. P. Huguley read a paper on "Ileus with Report of Cases," which will appear in the next issue of the Journal.

Dr. L. Sage Hardin said that the case showing spasm along several parts of the gut was unusually interesting and that it probably was of central origin. In this connection it is pertinent to inquire what causes the shock in the disease. There are the facies abdominalis, the low temperature and pulse and other indications of shock but ordinarily there is just a band around the intestines to account for the whole condition. It doesn't seem enough to explain it. Experiments upon dogs go to show that it is due in part to loss of fluid from the body. Proctoclysis relieves this in part.

In gangrenous cases enterostomy permits a by-pass and may save a life.

Dr. R. R. Kime said that the prevention of ileus was of great importance and that it lay in the careful handling of the intestines at the time of operations. There should be no pulling upon them and never should they be allowed to touch the skin. When there is abscess, it is unwise to manipulate extensively to void the pus. One had better drain and wait. Pituitrin is of value in inducing early peristalsis after abdominal operations thus preventing adhesions. Proctocysis is most important and should be used promptly whenever indicated.

Dr. E. C. Cartledge said that Babcock had reported two cases of spontaneous movement of the bowels under spinal anaesthesia and that this method had been subsequently used for the purpose of removing obstructions.

Dr. W. A. Selman said that he had seen Huguley's case of intermittent collapse of the intestine and that there were no adhesions, restrictive bands or other apparent cause for the trouble. He had two cases of spasm in children when he had to use bromides to lessen the nervous activity before he could get the purgatives to act.

Huguley said in closing that he had had another case of collapsed intestine in which nothing could be forced through the lumen. The sides were adherent internally. Pellagra changes in the intestines are similar to this.

Dr. R. R. Kime reported a case of double vagina and double uterus. The partition between the two vaginae was flaccid and lay readily to one side or the other so as to be readily missed in examination. The patient had miscarried at second month and curettage did not show much, but on the second day the gauze left in the womb could not be found. Another search revealed it and the cause of the trouble. The wrong vagina had been entered.

Dr. Arch Smith said that he had had a similar case in which there had been miscarriage and confusion of the vaginal passages, leading to a double uterus.

Dr. G. M. Niles read a paper on "A Study in Intestinal Peristalsis with Illustrations." It appears elsewhere in the Journal.

Dr. A. B. Elkin said that the recent advances in x-Ray work has resulted in fine pictures of the abdomen taken laterally as well as anteroposteriorly and this gives us a true concept of the condition. The different forms of peristalsis can thus be demonstrated.

Dr. Derr said that Niles deserves great credit for the study he has made of peristalsis, but he does not entirely agree with him that the kinks shown call for operation. Kinks at the hepatic flexures often make trouble that is referred to the appendix and operated for on that account. An x-Ray would show the truth.

Dr. E. C. Thrash said that tuberculous ulcers of the intestines often cause obstruction and great pain. Fistulae result and they are incurable. They are below the peritoneum, but are inoperable except as to drainage.

Niles said that Derr had misunderstood him in part. The point is that the mere fact of seeing things out of place or shape does not in itself call for operation, but a careful study of them in each instance will show what is to be done. Besides, our ideas of the topography of the abdomen must be revised and changed from those the text books have been teaching.

